# Single-Cell Analysis Reveals Transcriptomic Reprogramming in Aging Cardiovascular Endothelial Cells

**DOI:** 10.3389/fcvm.2022.900978

**Published:** 2022-05-09

**Authors:** Bo Gou, Xiaojing Chu, Yi Xiao, Pinxuan Liu, Hao Zhang, Zeyu Gao, Moshi Song

**Affiliations:** ^1^State Key Laboratory of Membrane Biology, Institute of Zoology, Chinese Academy of Sciences, Beijing, China; ^2^Beijing Institute for Stem Cell and Regenerative Medicine, Beijing, China; ^3^Institute for Stem Cell and Regeneration, Chinese Academy of Sciences, Beijing, China; ^4^University of Chinese Academy of Sciences, Beijing, China

**Keywords:** senescence, cardiovascular endothelial cells, scRNA-seq, age-dependent genes, transcriptomic reprogramming

## Abstract

The senescence of cardiovascular endothelial cells (ECs) is a major risk factor in the development of aging-related cardiovascular diseases. However, the molecular dynamics in cardiovascular EC aging are poorly understood. Here, we characterized the transcriptomic landscape of cardiovascular ECs during aging and observed that ribosome biogenesis, inflammation, apoptosis and angiogenesis-related genes and pathways changed with age. We also highlighted the importance of collagen genes in the crosstalk between ECs and other cell types in cardiovascular aging. Moreover, transcriptional regulatory network analysis revealed *Jun* as a candidate transcription factor involved in murine cardiovascular senescence and we validated the upregulation of *Jun* in aged cardiovascular ECs both *in vitro* and *in vivo*. Altogether, our study reveals the transcriptomic reprogramming in the aging murine cardiovascular ECs, which deepens the understanding of the molecular mechanisms of cardiovascular aging and provides new insights into potential therapeutic targets against age-related cardiovascular diseases.

## Introduction

Senescence leads to a decline in cardiovascular function over time. Endothelial cells (ECs), pericytes, and vascular smooth muscle cells (vSMCs) are essential components of vessels for the cardiovascular system (1). Senescent ECs show changes in morphology, including an increase in size, polymorphic nuclei, flattening and vacuolization (2). It is well known that cardiovascular ECs are involved in the pathologies of age-related cardiovascular diseases (CVDs) (3, 4) and cardiovascular EC senescence has been identified as a major risk factor for the onset and progression of CVDs (5). Despite the importance of cardiovascular EC senescence in aging-related diseases, very limited studies have been focusing on the molecular dynamics of the aging cardiovascular ECs (6, 7). Accordingly, a comprehensive study across multiple time points may better illustrate the transcriptomic reprogramming in cardiovascular EC aging process.

Single-cell RNA sequencing (scRNA-seq) has enabled the detailed illustration of transcriptional characteristics at the single-cell resolution, serving as a highly powerful tool for depicting the unknown cell type-specific transcriptional landscapes and dynamic cellular communications in various tissues under physiological and pathological conditions (8, 9). As such, the establishment of large-scale scRNA-seq databases characterizing cell type-specific gene expression across the lifespan provides rich resources to carry out an in-depth study on the aging effects in certain tissues or cell types (10).

In this study, we reanalyzed a published multi-time point, single-cell transcriptomic dataset of murine organs (10), in which cell-specific aging effects were characterized in 23 mouse organs. Our study has a better focus on the cardiovascular ECs and presented molecular hallmarks of cardiovascular aging. We showed that the dynamic transcriptomic changed with age and identified hundreds of aging-related genes in cardiovascular ECs which were enriched in ribosome biogenesis, apoptosis, inflammation and angiogenesis-related pathways. We also characterized the cell-cell communications between ECs and other cell types during cardiovascular aging. Moreover, we identified *Jun* as a potential key regulator involved in murine cardiovascular senescence via transcriptional regulatory network analysis and further confirmed the upregulation of *Jun* in aged cardiovascular ECs both *in vitro* and *in vivo*. Altogether, our work depicts the transcriptomic landscape of murine cardiovascular EC aging, which has important implications for better understanding the molecular fingerprints of cardiovascular aging and contributes to new prospective therapies for age-related CVDs.

## Materials and Methods

### Cell Clustering of scRNA-seq Data

The scRNA-seq data were collected from a dataset of 23 organs from mice at six-time points after birth [1, 3, 18, 21, 24, and 30 months (m)], which was established by the Tabula Muris Senis Consortium (10). Seurat (v4.0.3) R package (11) was used to analyze the expression matrix. Heart and aorta data were firstly extracted and processed described as following. The “NormalizeData” function via the “LogNormalize” method and a scale factor of 10,000 were applied for data normalization; 2,000 highly variable genes were identified by the “FindVariableGenes” function *via* the “vst” method. Next, the data were scaled and the first 50 principal components (PC) from principal component analysis (PCA) were used to construct a Shared Nearest Neighbor (SNN) Graph. The “FindClusters” function was used to perform the graph-based clustering and the resolution was set to 0.5. Finally, the “RunTSNE” function was used with the top 50 PCA dimensions as input for visualization. Next, we extracted the EC data. In order to reduce the deviation in the sampling of the original data, in which cells from 30 months were dominant, we calculated the average cell numbers of the other time points, kept the same number of 30 m cells for the downstream analyses and redid the clustering with the same settings, but with 30 PC and resolution set to 1. Based on the clustering result, a sample was identified as outlier and removed, leaving 10 samples in the downstream analysis, including one sample of 1 and 3 m, respectively, and two samples for each of the other time point. The cells of the 10 samples were re-clustered using the same settings and approaches mentioned previously, and eight clusters were acquired.

### Gene Set Score Analysis

The senescence-associated secretory phenotype (SASP) gene set was downloaded from https://maayanlab.cloud/Harmonizome/ (12) with 75 genes identified in humans. Among them, 45 genes were matched to mouse genes and measured in the EC dataset. The “AddModuleScore” function from Seurat (v4.0.3) was used to calculate the SASP gene set score between different time points. We then applied a Wilcoxon test between the scores of 3 m and other groups and visualized the results using a ggpubr package (v0.4.0) (https://rpkgs.datanovia.com/ggpubr/).

### Differential Expression Analysis and Age-Dependent Gene Identification

The differentially expressed genes (DEGs) between the young group (3 m) and aged groups (18, 21, 24, and 30 m) were identified using the “FindMarkers” function in Seurat (v4.0.3) with the default Wilcoxon rank-sum test. *P*-values were adjusted using the Bonferroni correction to indicate significance. A regression function from an R package Monocle3 (v1.0.0) (13) was applied to identify genes changing with ages and *p*-values were adjusted using the Benjamini and Hochberg method to get the *q*-values. Genes with *q*-values <0.05 were considered as age-dependent genes (ADGs) and were then clustered using an R package Mfuzz (v2.52.0) (14). The expression profile of each cluster was visualized using the R package pheatmap (v1.0.12) (https://cran.r-project.org/).

### GO and Gene Set Enrichment Analysis

The “EnrichGO” function from R package clusterProfiler (v4.0.2) (15) was applied for GO enrichment analysis to show the over-represented pathways and GO terms of the genes. Results were then visualized with an R package ggplot2 (v3.2.1) (https://ggplot2.tidyverse.org). Gene set enrichment analysis was performed using a software GSEA (v4.2.1) (16) with the gene sets from Gene Ontology collection (c5.all.v7.2) and the permutation number was set to 2,500. FDR <0.25 and the absolute NES value > 1.5 were considered as significant.

### Cell-Cell Communication Analysis

CellPhoneDB (v0.22) (17) was used to perform cell-cell communication analysis with normalized counts of ADGs and cell type annotations used as the input data. Genes expressed in <10% of cells were removed in the analysis. Mean expression of each ligand-receptor pair was then calculated between EC and other cell types, and the pairs with *p*-values <0.05 were considered in cell-cell communication prediction.

### Transcriptional Regulatory Network Analysis

A SCENIC pipeline (v1.2.4) (18) with default parameters was applied on ADGs to identify the potential key transcriptomic regulators during aging. Normalized expression data of ADGs at each time point was used as input to construct a co-expression regulatory network. The network of transcription factor and its target genes was visualized with Cytoscape software (v3.8.0) (19).

### Animal Experiment

The experiment involving animals was performed in accordance with the Guidelines approved by the Institutional Animal Care and Use Committee at the Institute of Zoology, Chinese Academy of Sciences (CAS). C57BL/6J mice were housed at ~25°C with a 12 h/12 h light/dark cycle. Three male mice of 3 months (3 m) and three male mice of 22 months (22 m) were used for mouse aorta isolation.

### Cell Culture and Senescence Induction

Human umbilical vein endothelial cells (HUVECs) (20) were cultured in the endothelial growth factor medium EGM™2 with BulletKit™ (Lonza, CC-4176) supplemented with 2% fetal bovine serum (FBS) under standard culture conditions (37°C, with 5% CO2) on 0.1% Gelatin-coated plates. Cells within 10 passages were used for all the experiments. HUVECs were treated with 0.2 μM doxorubicin (Dox) for 48 h to induce cellular senescence.

### Quantitative PCR

Total RNAs of mouse aorta and HUVECs were extracted using TRIzol Reagent (Thermo Fisher Scientific, Waltham, MA, USA) and RNA-Quick Purification Kit (Real-gen Biotechnology, RN001) respectively, according to the manufacturer's instructions. For cDNA synthesis, 2 μg of total mRNA was reverse-transcribed into cDNA with RevertAid First Strand cDNA Synthesis Kit (Thermo Scientific, K1622). qPCR reactions were conducted in 96-well plates on a LightCycler 96 (Roche) using TB Green^®^ Premix Ex Taq™ (Takara, RR420A) according to the manufacturer's instructions. GAPDH or 18S rRNA of the same species was used as the endogenous control for normalization. The qPCR primers are listed in Supplementary Table 1. The relative gene expression data were calculated using the ΔΔCt method. The Student's *t-*test was applied to evaluate the difference between groups, and a *p*-value <0.05 was considered to be significant.

### SA-β-Gal Staining

Senescence was detected by Senescence β-Galactosidase Staining Kit (Beyotime, C0602) according to the manufacturer's instructions. In brief, HUVECs were washed with PBS, fixed in fixation buffer at room temperature for 15 min and washed three times with PBS for 3 min each time. The cells were then incubated in a freshly prepared SA-β-gal staining buffer at 37°C overnight. The images were then taken using a microscope (Olympus) and the percentages of senescent cells were calculated (the number of SA-β-gal-positive cells divided by that of all the cells in three random fields). *P*-value was calculated using Student's *t-*test to compare differences between different groups and a *p*-value <0.05 was significant.

## Results

### The Transcriptomic Landscape of Cardiovascular ECs Across Mouse Lifespan

**Figure 1:**
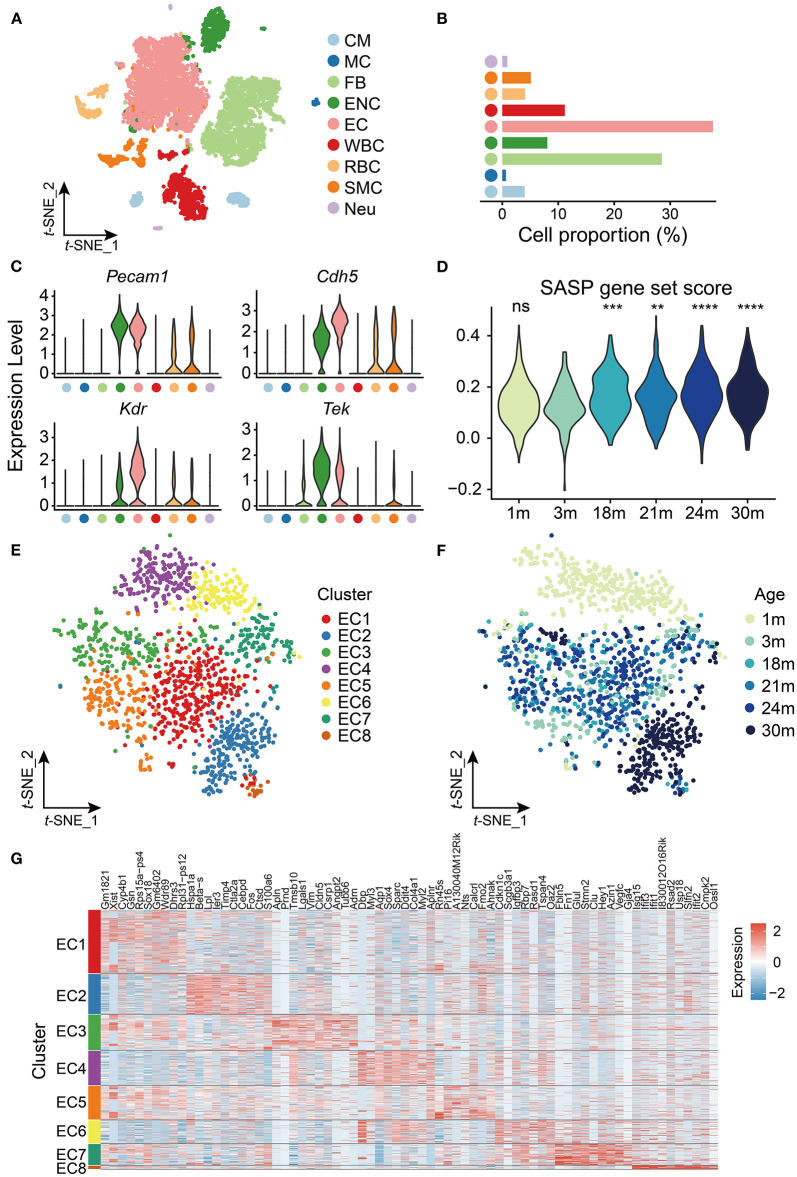
scRNA-seq analysis of cardiovascular endothelial cells (ECs) during aging. **(A)** *t*-SNE plot showing nine cell types from heart and aorta. CM, cardiomyocyte; MC, mast cell; FB, fibroblast; ENC, endocardial cell; EC, endothelial cell; WBC, leukocyte; RBC, erythrocyte; SMC, smooth muscle cell; Neu, cardiac neuron. **(B)** Bar plot showing cell proportion of nine cell types from heart and aorta. **(C)** Violin plots showing expression of EC markers in the heart and aorta. **(D)** Violin plot showing the SASP gene set score is significantly increased with age in ECs. ns, not significant; **p < 0.01; ***p < 0.001; ****p < 0.0001. **(E)** *t*-SNE plot showing eight clusters of ECs from heart and aorta. **(F)** *t*-SNE plot showing ECs from heart and aorta across the lifespan. **(G)** Heatmap showing top 10 markers expressed in each cluster of ECs from the heart and aorta.

To explore age-related transcriptomic signatures in the mouse heart and aorta, we extracted the transcriptome data of the heart and aorta from a published scRNA-seq dataset (10). Clustering analysis revealed nine cell types including cardiomyocytes (CMs), mast cells (MCs), fibroblasts (FBs), endocardial cells (ENCs), endothelial cells (ECs), leukocytes (WBCs), erythrocytes (RBCs), smooth muscle cells (SMCs) and cardiac neurons (Neus) (Figure 1A). Among them, ECs had the largest proportion (38%, Figure 1B) and highly expressed EC markers such as *Pecam1, Cdh5, Kdr*, and *Tek* (Figure 1C).

Considering the importance of ECs in cardiovascular aging, we further investigated the transcriptomic characteristics and reprogramming of cardiovascular ECs with age. We focused on the EC data of the heart and aorta, which contained 1,441 cells in total from six time points [1, 3, 18, 21, 24, and 30 months (m)] across the lifespan. The cellular senescence of cardiovascular ECs was evaluated at the six time points by scoring senescence-associated secretory phenotype (SASP), which is characterized by the increased expression and secretion of several cytokines (21), and we found that SASP expression increased in cardiovascular ECs from 18 months (Figure 1D).

We then performed clustering analysis on the cardiovascular ECs and identified eight clusters (Figure 1E) with different gene markers (Supplementary Table 2). As shown in Figure 1F, cardiovascular ECs of 1 m were specifically enriched in clusters EC4 and EC6, and ECs of 30 m were enriched in cluster EC2 (*p* < 0.05, Fisher's exact test), which was separated from ECs of other time points. Notably, embryonic development and/or circadian rhythm-related transcription factors *Dbp* and *Sox4* (22, 23) were specifically expressed in cardiovascular ECs of 1 m and were silenced in mature adults and elders. Conversely, *Lpl* that encodes lipoprotein lipase (24) was highly expressed in cardiovascular ECs of 30 m. Therefore, our observation agrees with the inextricable association between lipid metabolism and aging (25). Furthermore, apoptosis-related gene *Ier3* (26) was also highly expressed in aged cardiovascular ECs (30 m), suggesting that the increase of apoptosis was also involved in cardiovascular aging (Figures 1F,G). To sum up, cardiovascular ECs have distinct expression patterns at different time points across mouse lifespan.

### Differential Gene Expression Analysis Reveals Key Genes and Pathways in Aging Cardiovascular ECs

**Figure 2:**
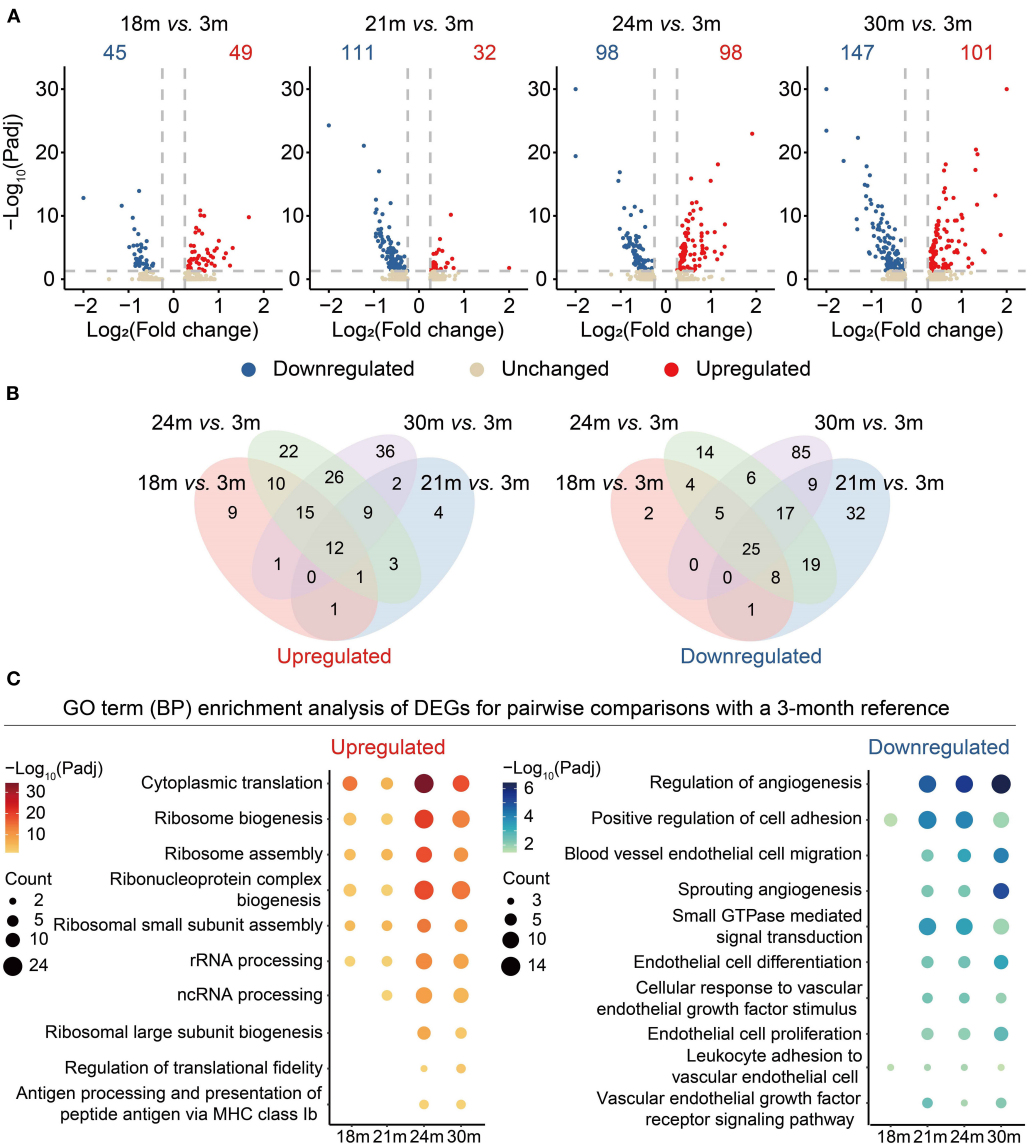
Differential gene expression analysis of cardiovascular ECs across the lifespan. **(A)** Volcano plots showing differentially expressed genes (DEGs) between 18 and 3 m, 21 and 3 m, 24 and 3 m, 30 and 3 m (from left to right), respectively. X-axis indicates the Log_2_(fold change); Y-axis indicates the –Log_10_(adjusted *p*-values). Upregulated genes are colored in red and downregulated genes are colored in blue. **(B)** Venn diagrams showing the numbers of overlapped upregulated (left) and downregulated (right) DEGs between four comparisons, including 18 vs. 3 m (red), 21 vs. 3 m (blue), 24 vs. 3 m (green), 30 vs. 3 m (purple). **(C)** Dot plots showing representative GO terms enriched in DEGs based on functional enrichment analysis. –Log_10_(adjusted *p*-values) are indicated by colors, and gene counts of each GO term are indicated by the dot size.

To better understand the genes changing in the aging process, we performed differential expression analysis for different age groups by taking 3 m (mature adult phage) as reference (Supplementary Table 3). As shown in Figure 2A, the number of differentially expressed genes (DEGs) gradually increased from 18 to 30 m when compared with 3 m, suggesting that aging was characterized by an accumulation of transcriptomic changes instead of a sudden shift. To identify the genes with potentially long-lasting effects from 3 to 30 m, we further interrogated the DEGs shared between all the comparisons and with consistent directions. A total of 12 genes were consistently upregulated while 25 genes were downregulated with age (Figure 2B; Supplementary Table 4). For instance, *APOLD1* that plays a role in the regulation of endothelial cell signaling and vascular function (27) was upregulated during aging. A long noncoding RNA *MALAT1*, a transcriptional regulator of cell migration (28) and cell cycle (29), was downregulated in aged cardiovascular ECs. Likewise, *Itgb1* was also downregulated during aging, consistent with a previous observation that the depletion of *Itgb1* in ECs results in impaired growth of blood vessels and reduced cardiac function (30).

Subsequently, by functional enrichment analysis on the gene sets changing with age, we investigated biological processes that were particularly affected by aging. We noticed that “ribosome biogenesis” (representative genes including *Rps17* and *Rps25*) was enriched in the upregulated DEGs, suggesting that a disorder of the ribosome dynamics might be involved in the aging process (Figure 2C and Supplementary Figure 1). Consistent with a previous study (31), downregulated genes were also enriched in the functions related to angiogenesis (representative genes including *Kdr* (also known as *VEGFR2*) and *Flt1*) and EC migration processes, suggesting that *VEGFR2* depletion in aging ECs may serve as one risk factor contributing to the reduction of the regenerative capacity during cardiovascular aging.

**Figure 3:**
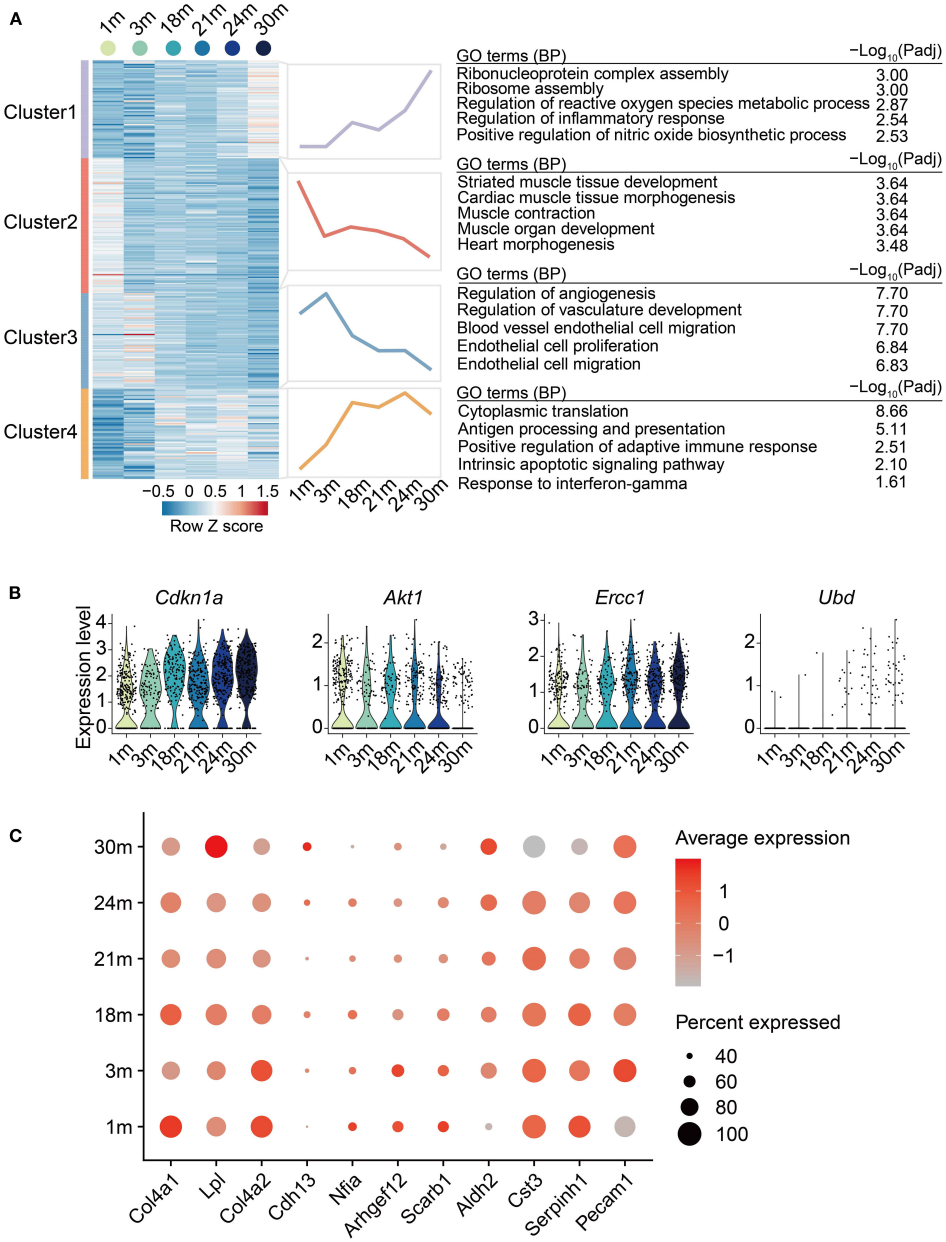
Age-related transcriptional dynamics in cardiovascular ECs. **(A)** Left: Heatmap showing expression signatures of age-dependent genes (ADGs) of ECs from heart and aorta with age; row-scaled Z scores are indicated by colors from blue (low) to red (high). Middle: Line plots showing average expression value of each cluster. Right: representative GO terms. **(B)** Violin plots showing the expression level of the overlapped genes between identified ADGs and genes acquired from GenAge database. **(C)** Dot plot showing the expression level of ADGs associated with cardiovascular diseases.

Next, to systematically discover gene sets whose expression level changed with age, we carried out a regression analysis on the EC single-cell data aforementioned and identified 329 age-dependent genes (ADGs, Supplementary Table 5). The ADGs were then classified into four clusters with distinct expression patterns (Figure 3A). The overall expression of Cluster 1 rose gradually with age after 3 m, with genes of ribosome assembly and inflammation-related pathways significantly enriched. Cluster 2 was enriched in muscle tissue development-related pathways, which showed a downregulation from 1 to 3 m, then reached a peak at 18 m, and was gradually downregulated afterward. Cluster 3 was upregulated in adulthood (from 1 to 3 m) and then downregulated in the later stages. The genes of this cluster were enriched in EC proliferation and biological processes related to angiogenesis, indicating that cardiovascular aging was accompanied by impaired EC proliferation and angiogenesis. Cluster 4 was continually upregulated with age till 18 m and maintained high expression through the entire elderhood, characterized by the activation of apoptotic signaling and immune-related pathways such as antigen processing and presentation. A total of four genes among the 329 ADGs of ECs were also reported as senescence-related genes in the GenAge database (https://genomics.senescence.info/genes/index.html) (32), including *Ubd* from Cluster 1, *Akt1* from Cluster 3, *Cdkn1a* (also called *P21*) and *Ercc1* from Cluster 4 (Figure 3B). In particular, *Cdkn1a*, a classic senescence marker gene, was upregulated with EC aging, which is in line with the observations made in the arteries of elderly individuals (33). Collectively, these results indicate that EC aging is accompanied with a series of transcriptional changes involving inflammatory-related processes, ribosome assembly, and disturbance of angiogenesis.

### Age-Dependent Genes Are Implicated in the Genetic Susceptibility of Age-Related Cardiovascular Diseases

To link the age-dependent genes with genetic susceptibility of age-related cardiovascular diseases, we intersected the disease-associated genes acquired from the GWAS Catalog database (March 2022) (34) with the identified 329 ADGs to illustrate the clinical relevance of EC aging effects. In total, 11 coronary artery disease-related genes were also identified as ADGs. These genes included *Cdh13* and *Lpl* from Cluster 1; *Nfia, Col4a1* and *Serpinh1* from Cluster 2; *Arhgef12, Col4a2, Scarb1* and *Cst3* from Cluster 3; *Pecam1* and *Aldh2* from Cluster 4 (Figure 3C), providing plausible molecular basis for the inextricable causal link between age and coronary artery disease. For example, in concert with the decreased expression of *Cst3* in aged mouse cardiovascular ECs, the mutant haplotype of *Cst3* gene (Cystatin C) has been associated with a lower plasma cystatin C concentration and a higher average number of stenoses per coronary artery segment in postinfarction patients (35). Furthermore, consistent with the continual upregulation by 18 m and maintained high expression during elderhood of *Aldh2* in mouse cardiovascular ECs, *ALDH2* transgenic mice showed accentuated myocardial remodeling and contractile dysfunction in aging (36). Taken together, our analysis presents the relevance between cardiovascular EC aging and the genetic susceptibility of cardiovascular diseases.

### Altered Ligand-Receptor Interactions Between Cardiovascular ECs and Other Cell Types During Aging

**Figure 4:**
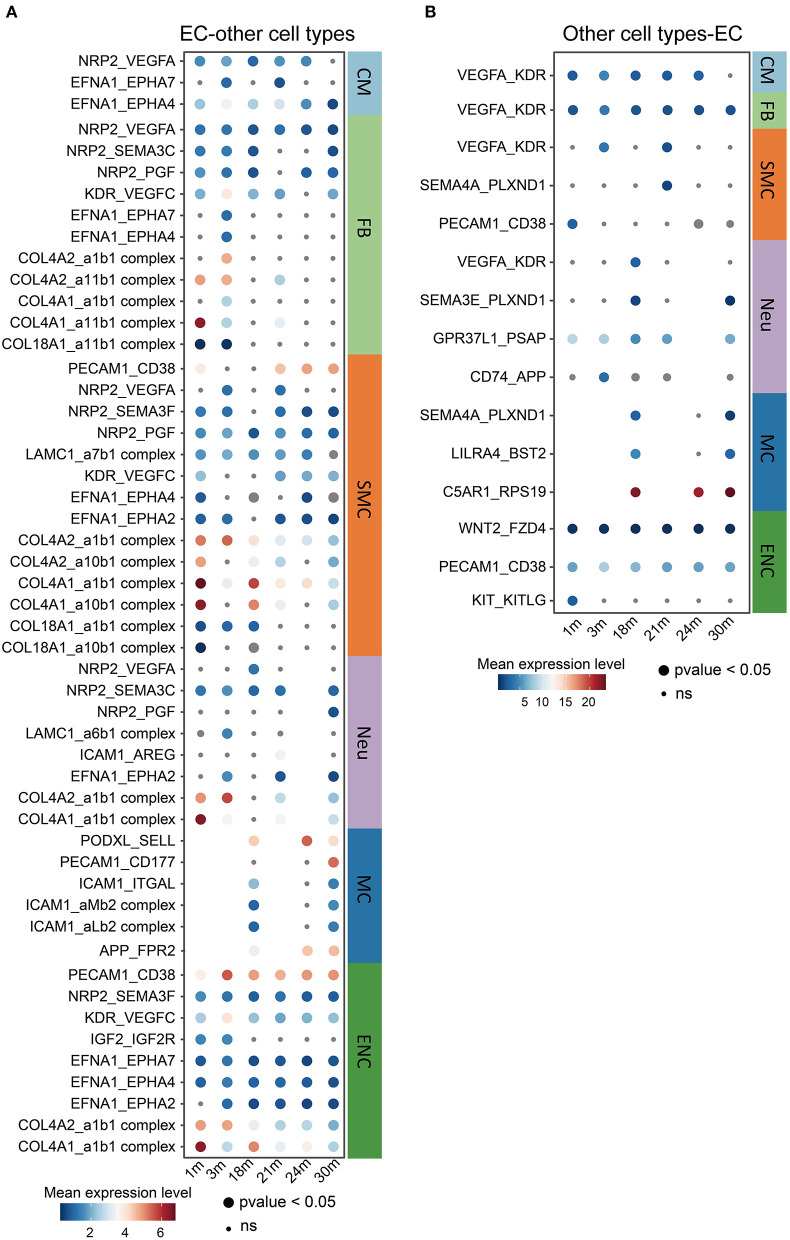
Changes in ligand-receptor interactions between cardiovascular ECs and other cell types during aging. **(A,B)** Changes of selected ligand-receptor interactions between ECs and other cell types (**A**: ECs express the specific ligand. **B**: receptor) during aging. CM, cardiomyocyte; MC, mast cell; FB, fibroblast; ENC, endocardial cell; EC, endothelial cell; WBC, leukocyte; RBC, erythrocyte; SMC, smooth muscle cell; Neu, cardiac neuron. Dot size represents the *p*-value. The color key indicates the mean expression level of each interacting pair.

It is known that the crosstalk between cardiovascular ECs and other cell types may play critical roles in the aging process and the pathology of aging-related diseases (37). For this reason, we performed a cell-cell communication analysis (Figures 4A,B) of all cell types found in the heart and aorta in the aforementioned dataset. Notably, many collagen genes, such as collagen type IV genes *COL4A1* and *COL4A2* (38), as ligands released by ECs to multiple other cell types including FBs, SMCs, Neus and ENCs, decreased during aging (Figure 4A). This is consistent with the previous findings reporting that the mutations in *COL4A1* and *COL4A2* may cause blood vessel abnormalities (39, 40), and that a CVD risk genetic factor rs4773144 is associated with the reduced expression of *COL4A1* and *COL4A2* gene in ECs (39, 41). These results imply that EC is a causal cell type of CVDs in which collagen type IV may play important roles in crosstalking with other cardiovascular cell types, thus pointing to the collagen genes as potential therapeutic targets in age-related CVDs. Interestingly, fewer communications, in which ECs were receptor cells, were observed compared to where they were sending cells (Figure 4B). Altogether, these observations highlight that ECs may mediate the aging process in other cell types by regulating cellular signaling via intercellular communications.

### Transcriptional Regulatory Network Analysis and Experimental Validation Uncover *Jun* as a Regulator of Cardiovascular Aging

**Figure 5:**
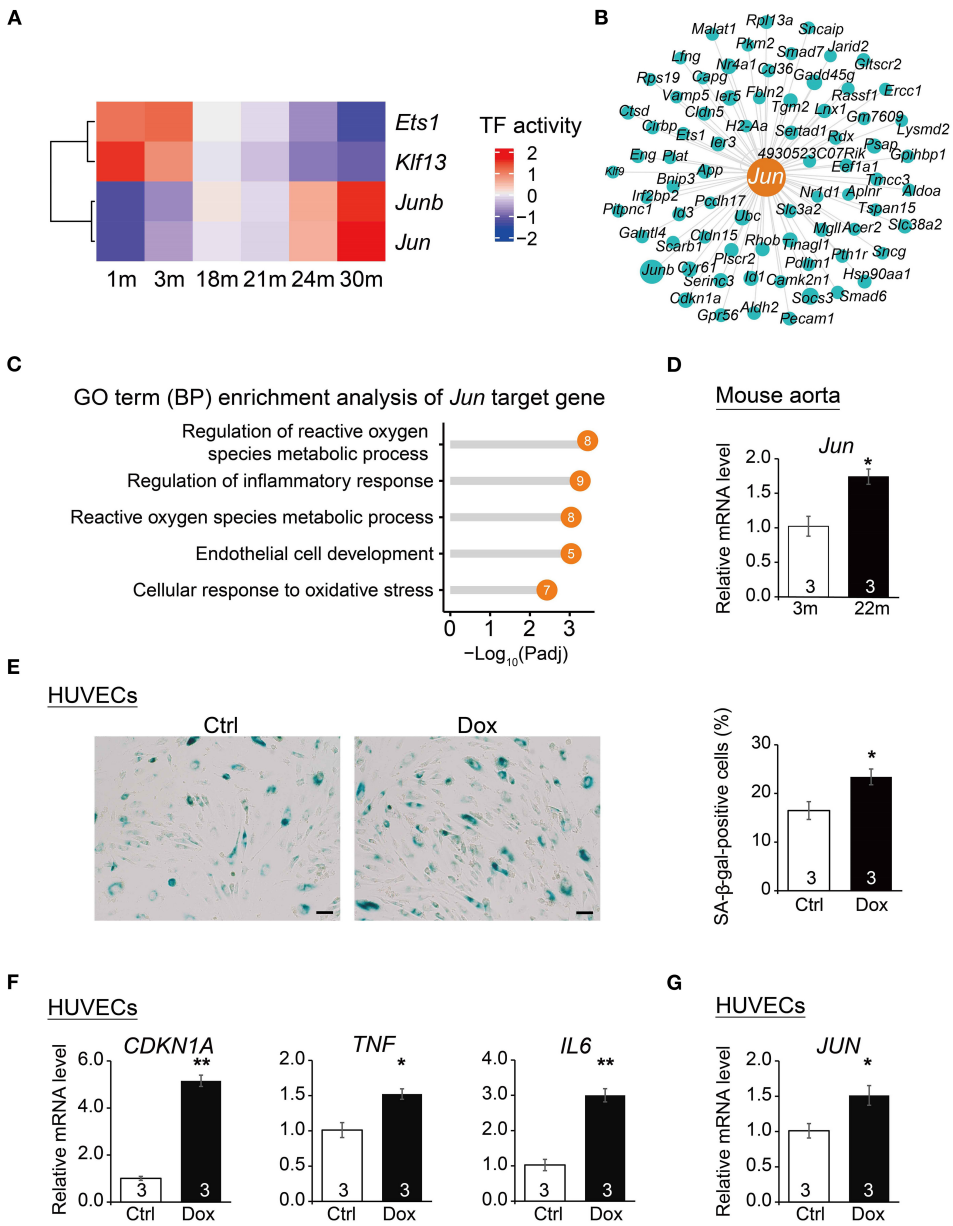
Transcription regulatory network analysis uncovers *Jun* as a regulator of vascular aging. **(A)** Heatmap of transcription factor (TF) activities at six time points. **(B)** Gene network showing target genes of *Jun*. The dot size presents relative weight values in the regulatory network. **(C)** Lollipop chart showing enriched GO pathways of *Jun* target genes. **(D)** qRT-PCR analysis of *Jun* mRNA levels in young (3 m) vs. aged mouse (22 m) aorta. Data are presented as the mean ± SEM (*n* = 3) biological repeats. *p < 0.05 (Student's *t-*test). **(E)** SA-β-gal staining in HUVECs treated with vehicle (Ctrl) or Dox (doxorubicin). Data are presented as the mean ± SEM (*n* = 3 independent experiments). *p < 0.05 (Student's *t-*test). Scale bar, 50 μm. **(F)** qRT-PCR analysis of mRNA levels of aging markers in HUVECs upon Dox treatment. Data are presented as the mean ± SEM (*n* = 3 independent experiments). *p < 0.05, **p < 0.01 (Student's *t-*test). **(G)** qRT-PCR analysis of *JUN* mRNA levels in HUVECs upon Dox treatment. Data are presented as the mean ± SEM (*n* = 3 independent experiments). *p < 0.05 (Student's *t-*test).

To dissect the key transcription factors related to cardiovascular EC aging, we performed SCENIC analysis (18) to construct gene regulatory networks of transcription factors and their target genes and identified a series of transcription factors (TFs) implicated in the cardiovascular EC aging process (Figure 5A). In particular, angiogenesis-related transcription factor *Ets1*, endothelial proliferation regulator *Klf13*, and AP-1 transcription factor subunits *Jun* and *Junb* were identified as potential key EC aging regulatory factors (regulons). Among them, *Jun* has been reported to regulate angiogenesis and cardiovascular EC apoptosis (42, 43). In addition, pathway enrichment analysis also showed that *Jun* target genes (Figure 5B) were enriched in signaling pathways such as regulation of reactive oxygen species metabolic process and regulation of inflammatory response (Figure 5C).

To verify the increased expression of *Jun* in senescent cardiovascular ECs, we isolated the aorta from young and elderly mice (3 and 22 m, respectively) to perform the *in vivo* evaluation of the expression of *Jun* in mouse aorta with age. The expression of *Jun* in mouse aorta from old mice (22 m) was higher than that of young mice (3 m) (Figure 5D), suggesting the potentially important role of *Jun* in mouse cardiovascular aging. Notably, by extracting EC gene expression from a single-cell RNAseq data of human adult hearts (44), we showed that JUN had a significantly higher expression level in the ECs of elders than that of individuals in their middle ages (Supplementary Figure 2). Furthermore, we examined the expression of *Jun* in senescent HUVECs chemically induced by doxorubicin (Dox). Exacerbated cellular senescence of HUVECs upon Dox treatment was verified by an increased percentage of cells positive for senescence-associated β-galactosidase (SA-β-gal) staining (Figure 5E) and by the upregulation of aging markers including *CDKN1A* (45), *TNF* and *IL6* (46) *via* qPCR analysis (Figure 5F). More importantly, we detected the increased expression of *JUN* in these senescent HUVECs (Figure 5G), suggesting that *JUN* may also be implicated in human EC aging and likely contribute to human cardiovascular aging-related diseases. Lastly, we found that *Jun* was highly expressed in the human cardiovascular tissue based on the GTEx portal database (http://www.gtexportal.org/home/) (Supplementary Figure 3), which further supports the putative important roles of *Jun* in the human cardiovascular system. Altogether, these results suggest *Jun* as a potential transcription factor underlying cardiovascular disease and aging.

## Discussion

In this study, we reanalyzed a single-cell transcriptomic data of mouse across the lifespan and characterized the transcriptomic reprogramming in cardiovascular ECs during aging. We found a list of cardiovascular EC aging-related genes and pathways including ribosome biogenesis, inflammation, apoptosis and angiogenesis-related genes and pathways. We also showed that some of the ADGs we identified were overlapped with age-related CVD genes, suggesting the aging effect of cardiovascular ECs may be implicated in the genetic pathology of cardiovascular diseases. Moreover, we revealed collagen genes as important players in the crosstalk between ECs and other cell types in cardiovascular aging. Lastly, we identified *Jun* as a candidate key transcription factor involved in cardiovascular EC senescence and validated the upregulation of *Jun* both in aged mouse aorta and in senescent human ECs. Taken together, our study depicts the transcriptomic landscape of cardiovascular EC aging, offering new insights into the molecular mechanisms of cardiovascular aging and potential therapeutic targets against age-related cardiovascular diseases.

Previous studies have reported extensive transcriptional changes underlying the aging process of multiple tissues and organs (8–10, 47, 48). Likewise, we identified a list of differentially regulated genes and pathways implicated in aging cardiovascular ECs. In particular, we showed that cardiovascular EC aging was associated with accelerated ribosome biogenesis accompanied by upregulated rRNA processing process, which can be partially explained by that a crucial component of ribosome biogenesis is the processing of ribosomal RNA (rRNA) (49). This finding is consistent with the previous result that accelerated ribosome biogenesis promotes aging and impaired ribosome biogenesis extends *C. elegans* lifespan (50). In addition, our observation that cell proliferation and angiogenesis-associated genes were downregulated during aging indicates that the decline of regeneration ability of ECs is an important marker of cardiovascular aging, consistent with the reported impairment of angiogenesis implicated in aging-related vascular dysfunction (5). On the basis of our findings, future work is needed to discriminate the genes and pathways with causal changes with age.

The cell signaling was changed in aging cardiovascular ECs. Particularly, collagen genes decreased in elder ECs and as signaling ligand interacted with various receptors on the other cell types such as FBs. These results imply that cardiovascular EC is one of the “upstream” cell types contributing to the aging processes in other cardiovascular cell types and suggest that co-culture with other cells should also be considered in future experimental validation to better illustrate cardiovascular aging. Additionally, we identified *Jun* as a potential transcriptomic factor involved in the cardiovascular aging process. We observed a high activity of *Jun* in the aging aorta of mice and senescent HUVECs of human genetic background. *Jun* being an a component of the AP-1 (activator protein 1) transcription factor (51), our results suggest that *Jun* in ECs may also serve as an important regulator involved in cardiovascular aging, which agrees with the importance of AP-1 in the initiation and progression of age-related cardiovascular dysfunction and CVDs (52). Further work is required to construct a cardiovascular EC-specific *Jun*-knockout mouse model to explore whether *Jun*, when properly regulated, could serve as a therapeutic target to ameliorate cardiovascular aging.

Our study was to some extent limited by that only about 1,400 cardiovascular ECs from 10 mice were analyzed and the gender of mice from time points were not matched. Particularly, only one male mouse of 1 m and one female mouse of 3 m were included in our analysis, and the ECs from one mouse dominated the group of 30 m, which might bias the results by introducing donor/gender-specific effects as potential confounders. However, we observed that for each time point of 18-24 months, cells from the same group of mice were clustered together, suggesting no obvious donor effects in the data (Supplementary Figure 4). Our study has also advantaged in improving the understanding of the regulatory mechanism of cardiovascular EC senescence and age-related CVDs. Larger cell counts in future studies could better estimate the heterogeneity of cardiovascular ECs during aging. Overall, our work reveals the transcriptomic reprogramming in aging cardiovascular ECs by highlighting several important genes (such as *Jun* and collagen genes) and pathways (such as ribosome biogenesis and apoptosis), which provides new insights into the molecular mechanism of cardiovascular aging and potential therapies against human age-related CVDs.

## Data Availability Statement

Publicly available datasets were analyzed in this study. This data can be found at: https://figshare.com/articles/dataset/Processed_files_to_use_with_scanpy_/8273102/3.

## Ethics Statement

The animal study was reviewed and approved by Institutional Animal Care and Use Committee at the Institute of Zoology, Chinese Academy of Sciences (CAS).

## Author Contributions

MS conceived and designed the project. BG performed the bioinformatic analysis. XC supervised the single-cell transcriptomic analysis. YX and BG performed the cell experiments. ZG and HZ isolated the mouse aorta. YX, HZ, and PL performed RNA extraction and qPCR experiments. MS, BG, and XC interpreted the results and wrote the manuscript. All authors reviewed the manuscript.

## Funding

This work was supported by the National Key Research and Development Program of China (2020YFA0113400), Beijing Natural Science Foundation (JQ20031), the National Natural Science Foundation of China (81870228, 81922027, and 81921006), and the State Key Laboratory of Membrane Biology.

## Conflict of Interest

The authors declare that the research was conducted in the absence of any commercial or financial relationships that could be construed as a potential conflict of interest.

## Publisher's Note

All claims expressed in this article are solely those of the authors and do not necessarily represent those of their affiliated organizations, or those of the publisher, the editors and the reviewers. Any product that may be evaluated in this article, or claim that may be made by its manufacturer, is not guaranteed or endorsed by the publisher.
