# Supplementary material for: Single-Cell Analysis Reveals Transcriptomic Reprogramming in Aging Cardiovascular Endothelial Cells
Source: Front Cardiovasc Med. 2022 May 9;9:900978. doi: 10.3389/fcvm.2022.900978 (PMC9124831; doi:10.3389/fcvm.2022.900978)
Supplement: Supplementary file 6 [file Data_Sheet_1.docx]

Supplementary Materials

# Supplementary Figures

##
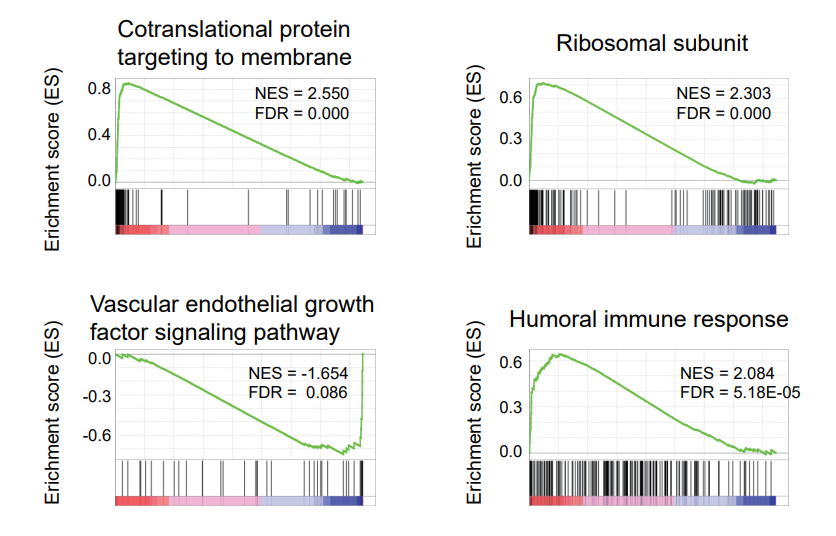


## Supplementary Figure 1. Function annotations of DEGs between 3m and 30m for ECs. Gene set enrichment analysis (GSEA) showing gene sets associated with EC aging.


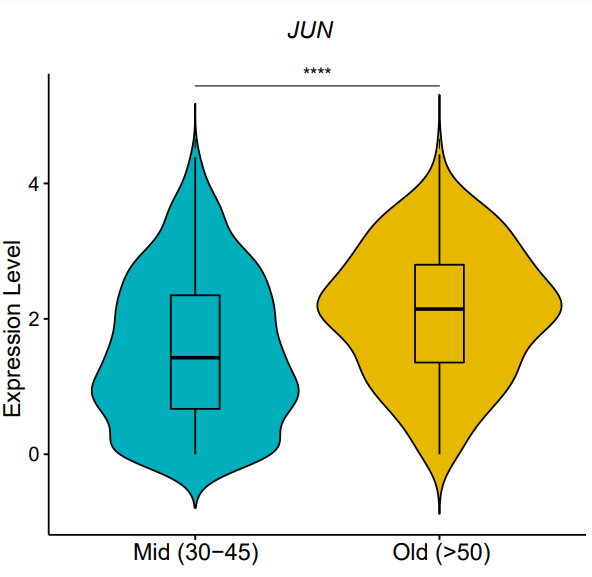


**Supplementary Figure 2.** **Relative expression of *JUN* in ECs of human adult hearts.** Violin plots combining boxplots showing the relative expression of *JUN* in ECs of human adult hearts. *****p* < 0.0001 (Wilcoxon test).


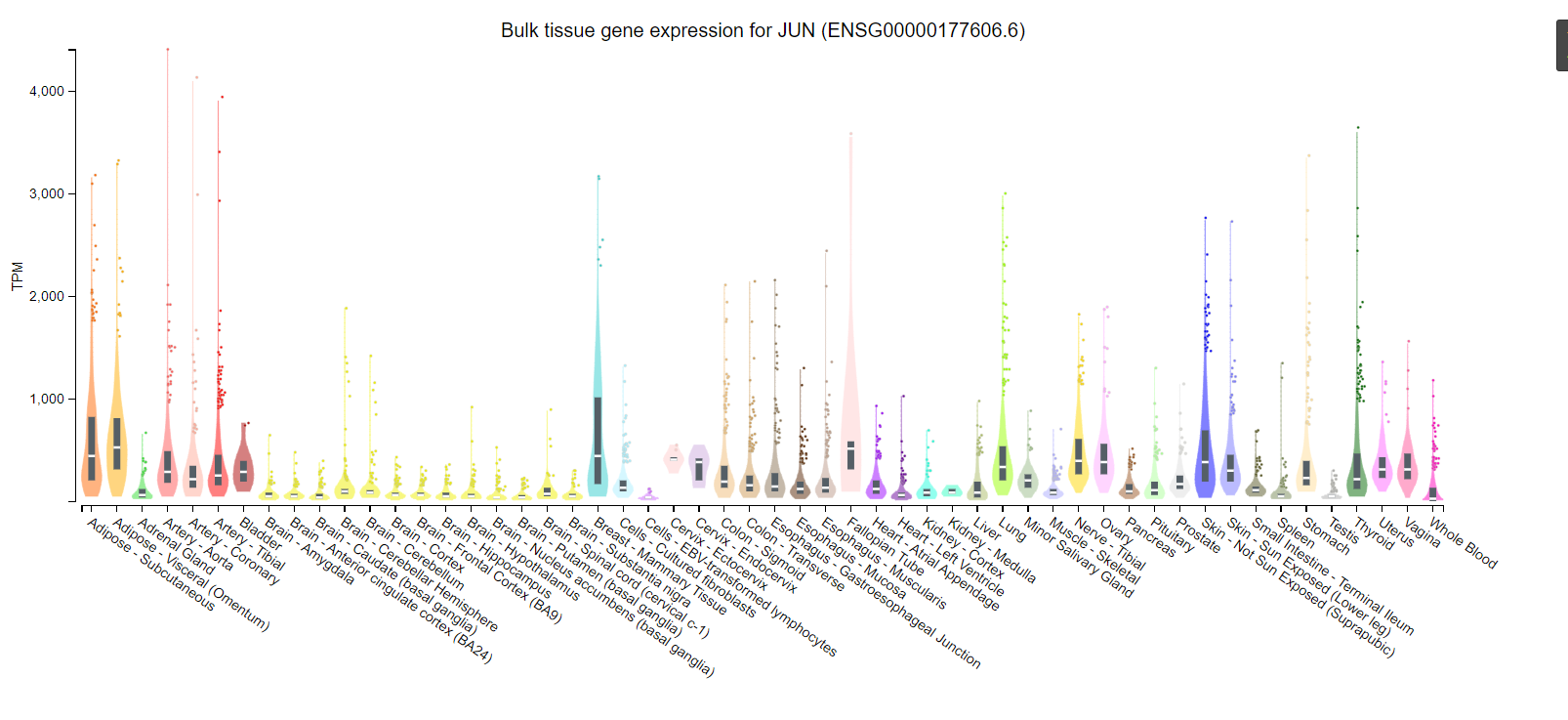
**Supplementary Figure 3.** **Expression characteristics of *JUN*.** Violin plot showing *JUN* expression level across various tissues obtained from the GTEx Portal (<https://gtexportal.org/>).


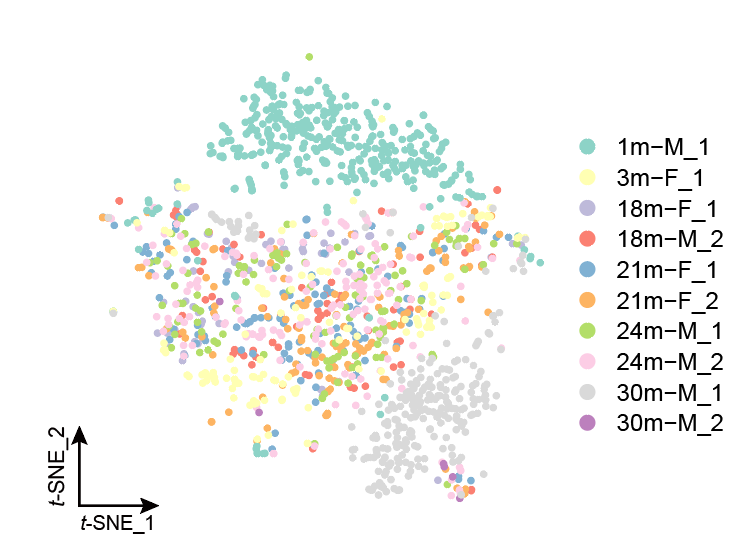


**Supplementary Figure 4.** ***t*-SNE plot showing the gene expression pattern of ECs across donors.** M, male; F, female. Different color represents all the mice used in the analysis.
